# Improvements in the Management of Trauma Patients With the Introduction of a Lower Limb Trauma Coordinator

**DOI:** 10.5812/kowsar.22517464.3424

**Published:** 2012-01-15

**Authors:** Dulani Mendis, Martin Vesely

**Affiliations:** 1 Department of ENT Surgery, Bimingham Children’s Hospital, Steelhouse Lane, Birmingham B4 6NH, England.; 2 Department of. Plastic Surgery, St George’s NHS Trust, London SW17 0QT, England.

**Keywords:** Lower Extremity, Trauma

## Abstract

Mortality and morbidity from trauma continues to be a serious, ubiquitous public health problem. Our short communication reports on the benefits of a dedicated lower limb trauma coordinator (LLTC) to the trauma service of a busy inner-London Plastics unit. This is based on a retrospective case-note based audit; performed 19 months prior to the introduction of the LLTC and for 16 months after. After the introduction of a LLTC our statistical analysis demonstrated a significant improvement in trauma timings in terms of injury to referral time, time to first plastics operation and duration of inpatient stay. This suggests the use of a fully qualified nurse with an orthopaedic background as a coordinator may prove to be highly advantageous over a non-clinical administrator improving the overall journey of the lower limb trauma patient in the English National Health Service.

## 1. Background 

Mortality and morbidity from trauma continues to be a serious, ubiquitous public health problem. The multidisciplinary approach to trauma involving multiple specialities, with differing standards of care prompted the publication of ‘Better Care for the Severely Injured’ (2000, revised 2003); a collaborative report by the Royal College of Surgeons and the British Orthopedic Association. This decisive report emphasises the importance of the standardisation of care, audit, and the development of a National Trauma Service with integrated systems and opportunities to facilitate a coordinated approach to injury (1). Following on from this, a later questionnaire study (Browne et al, 2006) has highlighted ongoing deficits in achieving the aforementioned standards of care particularly in relation to soft tissue standards. Most of the surveyed hospitals did not have plastic surgery available on site and the majority of delays were associated with organisational constraints (2).

## 2. Objectives

In consideration of these findings, we report on the benefits of a dedicated lower limb trauma coordinator (LLTC) to the trauma service of a busy inner-London plastic surgery unit.

## 3. Materials and Methods

Data collected via a retrospective case-note based audit; performed 19 months prior to the introduction of the LLTC and for 16 months after.

## 4. Results

After the introduction of a LLTC out of 157 cases, our statistical analysis demonstrated a significant improvement in trauma timings in terms of injury to referral time [P = 0.05; 95% Confidence Interval (CI) -29 to 0 days], time to first plastics operation (P = 0.0005; 95% CI: -24 to -7 days).

Statistical analysis: Parametric data with an independent t-test using the medcalc ® software program (http://www. medcalc.be)). A reduction in the transfer time of in-patients from other hospitals to our plastics department was also seen but this did not reach statistical significance (P = 0.07). The proportion of patients who were transferred to our unit having had their initial surgical procedure at another hospital rather than being transferred immediately also decreased, but this was not statistically significant (P = 0.34). In addition, in the second cycle of the audit we found the mean time from injury to definitive soft tissue cover to be 7.9 days (range 1 – 42). This aspect was not analyzed in the first cycle.

## 5. Discussion

Following on from published guidance, the management goals of treating open lower limb fractures include a combined approach with early assessment by experienced orthopaedic and plastic surgeons. Ideally, debridement, irrigation and fracture reduction/stabilisation should be achieved within six hours of admission. If plastic surgery input is likely to be required e.g. for significant soft tissue defects (usually Gustillo II fractures), then referral should be made within twenty-four hours (3). Definitive soft tissue cover should occur within 5 days of injury. Since the introduction of a LLTC we have been able to show a reduction in time from injury at all stages of the patient pathway for patients requiring soft tissue cover. This has enabled our department to achieve the recommendations set out in the published guidelines on lower limb trauma management for the majority of our patients (3). The reasons for this maybe multifactorial such as a greater awareness of the importance of early plastic surgical input in the management of complex lower limb trauma; better transfer arrangements so that patients are transferred with a management plan already in place and an appropriate theatre slot booked; better communication and coordination between the numerous specialities involved including orthopaedics, plastic surgery, microbiology, physiotherapy, dieticians and nursing staff and earlier and better discharge/rehabilitation arrangements. These lead to better logistical planning for these patients with fewer surgical procedures, allowing for faster treatment with the overall intention of fewer delays in care.

The use of a fully qualified nurse with an orthopaedic background as a LLTC proved to be highly advantageous over a non-clinical administrator; as additional abilities included:

1. Prioritizing patients according to clinical need;
2. Obtaining relevant clinical details particularly for patients from other units awaiting transfer;
3. Establishing a good rapport with the orthopaedic department;
4. Good knowledge and experience of the orthopaedic management of trauma;
5. Opportunities to educate other staff.

In addition, the LLTC provided a single point of contact for all staff involved in the management of trauma patients which facilitated communication and co-ordination of patient care. The organisation of patient transfer and additional operating lists, which are often necessary to accommodate trauma patients because of the unpredictability of the workload, is highly time-consuming, particularly in the current constraining financial climate. To ensure that patient care is not compromised by the inefficiencies of the system, a large part of this organisation falls to the junior doctors, taking them away from their training and service commitments.

A LLTC is not only able to streamline the delivery of care (in terms of timing of surgery) but can also assist junior doctors with a burdensome organisational task. We have not specifically done a cost analysis however the reduction in bed-days is likely to have caused a cost reduction, which alone may justify the salary and expenses of a LLTC.

In our plastic surgery unit, the introduction of a LLTC for the management of trauma patients has assisted junior doctors in their service commitments, improved team communication both within the department and between referring hospitals contributing to enhanced patient care by improving on trauma target times. Such a position may in fact prove to be cost-neutral to the hospital, however the potential benefits maybe applicable nationwide to all units providing a complex lower limb service.
